# Politics matter more than credentials in laypeople’s judgments of expertise

**DOI:** 10.1038/s41598-026-40053-0

**Published:** 2026-03-09

**Authors:** Mertcan Güngör, Nathan Ballantyne, Jared B. Celniker

**Affiliations:** 1 Department of Psychology, University of California - Irvine, Irvine, USA; 2 School of Historical, Philosophical and Religious Studies, Arizona State University, Tempe, AZ USA

**Keywords:** Cultural and media studies, Cultural and media studies, Psychology, Psychology

## Abstract

We explored the cues laypeople use to determine who is an expert on a given topic. In Study 1 (*n* = 208), we collected participants’ self-reports of cues they consider important when evaluating a putative expert. Across various topics, participants rated good cues (e.g., relevant degree, more experience) as more important for expertise while disregarding poor or superficial cues (e.g., appearance). In Study 2 (*n* = 498), we conducted a conjoint experiment where we manipulated several cues of expertise in biographies of hypothetical experts. In line with the self-reports, good cues had the strongest effects on trust. In Study 3 (*n =* 1,776), we examined whether participants would still recognize good cues when they received information about the expert’s political views. Using a highly politicized topic—abortion—we manipulated the expert’s views along with their credentials. Participants trusted likeminded experts more, and the effect of the expert’s views on participants’ trust was more than twice as large as the effect of credentials. Our results paint a complicated picture, where laypeople know what qualities to look for in experts but attend to political information more readily than to good cues of expertise.

**Supplementary Information:**

The online version contains supplementary material available at 10.1038/s41598-026-40053-0.

## Introduction

Faced with uncertainty about important questions, some people attempt to “do their own research”^1,2^. But knowing nearly anything interesting about the world requires depending on knowledgeable others. For millennia, humans have used division of labor and specialization to construct knowledge beyond what any person working alone could piece together over the course of their lifetime. When we are uncertain about a question but others know the answer, we benefit from the ability to identify the people in the know. We can call such people “experts”.

Identifying experts is an essential task but is easier said than done. The very idea of expertise is in the crosshairs in social and political conflicts. For better or worse, the idea of expertise is connected to the politicization of policy-relevant science as well as the distrust of institutions and elites. In fact, many recent observers have declared a “crisis of expertise”. That “crisis” has been taken to mean many things, including a decline of trust in established authorities and the emergence of “thought leaders”^3^ and “science influencers”^4^, the existence of rival claims to expertise without a settled way to decide amongst claimants^5^, the use of doubt-inducing strategies by corporations and elites to obscure scientific consensus from the general public^6^, and even the widespread public rejection of any meaningful distinction between the knowledgeable and the ignorant^7^.

Just like talk, people claiming expertise is cheap; we should be shrewd when evaluating such claims. But are there effective strategies for identifying experts? Researchers have noted that strategies are different for experts than for novices or laypeople. Experts, on the one hand, can calibrate others’ expertise by reference to their own—that is, they can leverage their knowledge to identify the knowledgeable^8,9^. On the other hand, novices, by definition, do not have expert-level knowledge. A minority view^10,11^ holds that novices are necessarily “blind” when trying to find experts and so are forced to exercise trust. The majority view, however, is that novices are not invariably “blind”. Novices can sometimes acquire special types of empirical evidence to help them identify expertise and even reasonably distinguish between rival experts.

Although there is no canonical view about what evidence novices should pay attention to when seeking expertise, theorists agree on some main ideas^9,12–14^. For instance, one standard idea is that experts’ experience—perhaps represented in the form of a “track record”—can be a cue for novices to recognize expertise. Another widely noted cue is credentials or certifications. And another cue is the opinions of known experts about a putative expert in question. Of course, not every type of evidence about experts happens to be good evidence—that is, the type of evidence that guides people to accurate judgment concerning the quality of someone’s expertise. Naturally, people can learn facts about experts’ height, weight, sex, ethnicity, and so on, but in many domains such facts are presumably poorly correlated to the quality of experts’ knowledge. Accordingly, such evidence typically constitutes a poor or misleading cue for the identification of expertise. We will rely on that intuitive, rough-and-ready distinction between *good cues* and *poor cues* in this article.

Can people attend to good cues when identifying and assessing expertise? There is little existing empirical evidence, but we have strong reason to believe that people can sometimes track good cues. Across many domains of everyday life, we expect people to behave reasonably when assessing expertise. Laypeople, including children, often trust the opinions of individuals who have more relevant expertise^15–19^—we are not surprised when a sick person goes to a doctor instead of a plumber. Laypeople often show skepticism toward implausible claims, even when the source of the claims has relevant expertise or holds a position of authority^20^. That broad kind of reasonableness should be unsurprising, given that people are generally epistemically vigilant^21^.

Of course, being reasonable sometimes doesn’t imply that people assess expertise perfectly. People might implicitly or explicitly identify cues that they sincerely believe are important but are in fact irrelevant for identifying expertise. For instance, people sometimes treat race and gender as indicative of scientists’ credibility^22^. In other cases, what are good cues in one situation can mislead elsewhere. For instance, having personal experience can be a good cue in most cases^17^, but one person’s experience may not be generalizable for topics where desired outcomes highly depend on individual variability (e.g., nutrition, skincare) or luck (e.g., stock market^23^. Another example is the phenomenon of “epistemic trespassing”^24^, which involves experts who stray from their actual domain of competence but nevertheless pass judgment. One case of epistemic trespassing features Linus Pauling, the Nobel Prize-winning chemist who later touted the power of megadoses of vitamin C to treat cancer. When a trespassing expert has impressive credentials or experience in a different domain, their background might easily mislead novices. Despite these imperfections, such errors in lay evaluation of expertise might be correctable through better education and more responsible behavior on the part of experts.

However, there’s reason to suspect that lay evaluations of expertise are subject to biases that are more difficult to overcome. Political partisans evaluate politically-tinged information in biased ways^25^—and we should expect that their judgments of expertise will be no different. Though some political biases might be more expressive than genuinely believed^26–28^, partisan reactions to the COVID-19 pandemic suggest that such biases are often sincere and costly. Despite substantial consensus amongst knowledgeable experts, Republicans trusted public health guidance less and died more because of it^29^. Many Republicans placed their trust in authorities with epistemologically weaker credentials, coinciding with the increase of many Americans treating “influencers” such as Joe Rogan as news sources^30^. While the response to public health guidance during the pandemic seems to paint a rosier picture of liberals’ evaluations of expertise, controlled experiments generally show that liberals and conservatives both display a similar tendency to evaluate scientific information in biased ways^31–33^. If partisans are prone to biased evaluations of expertise—not mere errors—then different interventions will be required to improve their expertise identification.

The current studies aim to address key empirical gaps in our understanding of how laypeople evaluate expertise. We examined the extent to which people weigh good and poor cues when deciding which expert to trust, and we tested whether their evaluations of expertise are skewed by political biases.

## Results

### Study 1

**Fig. 1:**
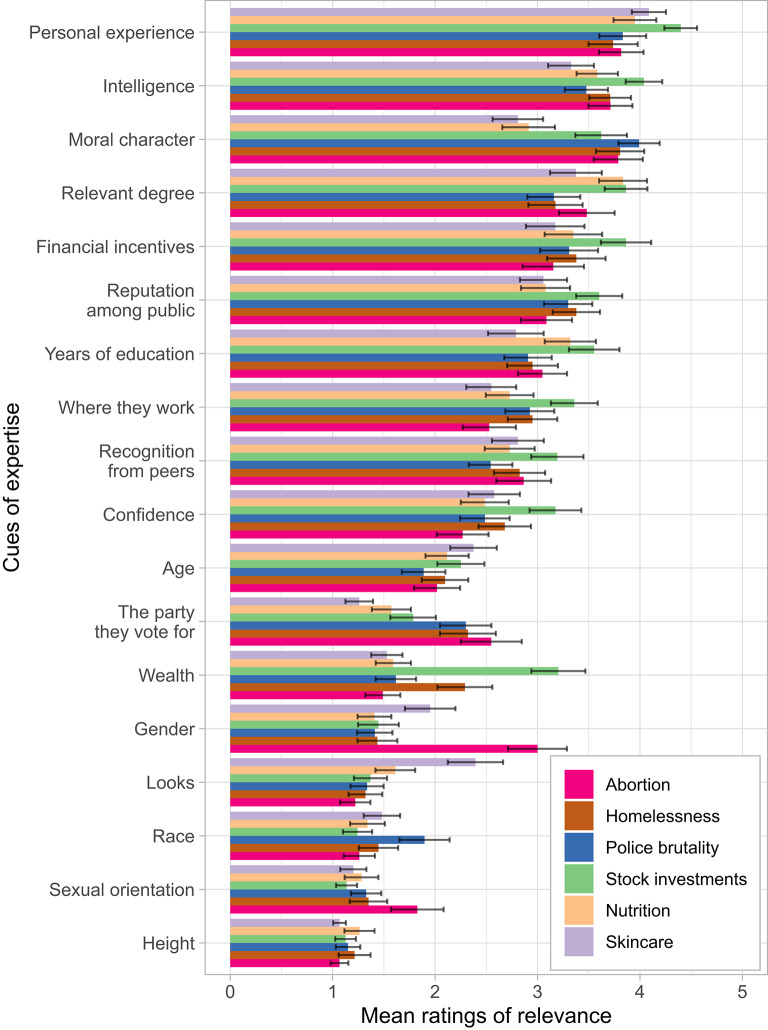
Self-reports of relevant cues (Study 1). Error bars represent 95% confidence intervals.

In Study 1, we asked participants (*n =* 208) which factors they thought were relevant when judging whether someone has expertise (Fig. 1). We asked about three politicized topics (abortion, homelessness, police brutality) and three non-politicized ones (nutrition, skincare, stock investments). Attributes that were rated by participants as the most relevant could be classified as “soft skills”—such as personal experience and intelligence—along with those that signal trustworthiness, such as moral character, financial incentives, and reputation among the general public. Those attributes were followed by domain-specific credentials that can be displayed more easily, such as years of education, having a degree relevant to the topic, and peer recognition in the form of awards or prizes.

Participants perceived some attributes as more relevant for expertise depending on the topic. For example, they placed greater importance on the expert’s political preferences when they perceived a topic as more politicized (*b* = 0.20, *SE* = 0.04, *p* <.001) and had stronger moral convictions about it (*b* = 0.11, *SE* = 0.04, *p* =.004). Liberals seemed to care slightly more about the expert’s politics but the relationship was not significant (*b* = −0.05, *SE* = 0.03, *p* =.069). In addition, some attributes were seen as relevant only for one topic but trivial for all others. For example, an expert’s wealth only mattered when getting advice on stock investments (*F*(5, 463.24) = 61.66, *η*^*2*^_*P*_ = 0.40, *p* <.001); the expert’s gender mattered for abortion (*F*(5, 471.97) = 55.15, *η*^*2*^_*P*_ = 0.37, *p* <.001); and the expert’s looks mattered for skincare (*F*(5, 455.99) = 39.36, *η*^*2*^_*P*_ = 0.30, *p* <.001).

### Study 2

Participants in our first study identified certain attributes of experts that they found relevant when determining who to trust. In Study 2, we tested whether the self-reported attributes actually impacted people’s trust toward experts using a conjoint experiment. Conjoint experiments are a tool for examining how people simultaneously evaluate multiple attributes that can introduce trade-offs (e.g., demographic characteristics vs. policy preferences of political candidates). Each factor is manipulated simultaneously for each participant, which allows researchers to capture the overall effect of each attribute by calculating the average marginal component effects (AMCEs)^34,35^. Unlike full factorial designs, conjoint experiments remain feasible (in terms of sample size and statistical power) as the number of factors increases. In Study 2, we manipulated six attributes that received moderate-to-high relevance ratings in Study 1, along with the expert’s gender, making a conjoint design more practical.

Participants (*n* = 498) read short biographies of hypothetical experts on the same six topics as the previous study. The expert either had a relevant or an irrelevant major, studied the topic for a long or a short time, studied at an Ivy League institution or a less famous one (e.g., Mayville State University), received many citations and awards from their peers or not, had recognition from the public or not, and had some anecdotal experience with the topic or not. Each attribute was independently randomized, meaning that the experts could be stronger in some respects but weaker for others. We measured the experts’ perceived trustworthiness, expertise, and credibility (see Methods). Even though the former two have been conceptualized as distinct constructs^36,37^, we aggregated the items due to the high degree of convergence among them (with *α*’s between 0.95 and 0.97 across topics); we will refer to the aggregate as “trust” for the sake of simplicity.

**Fig. 2:**
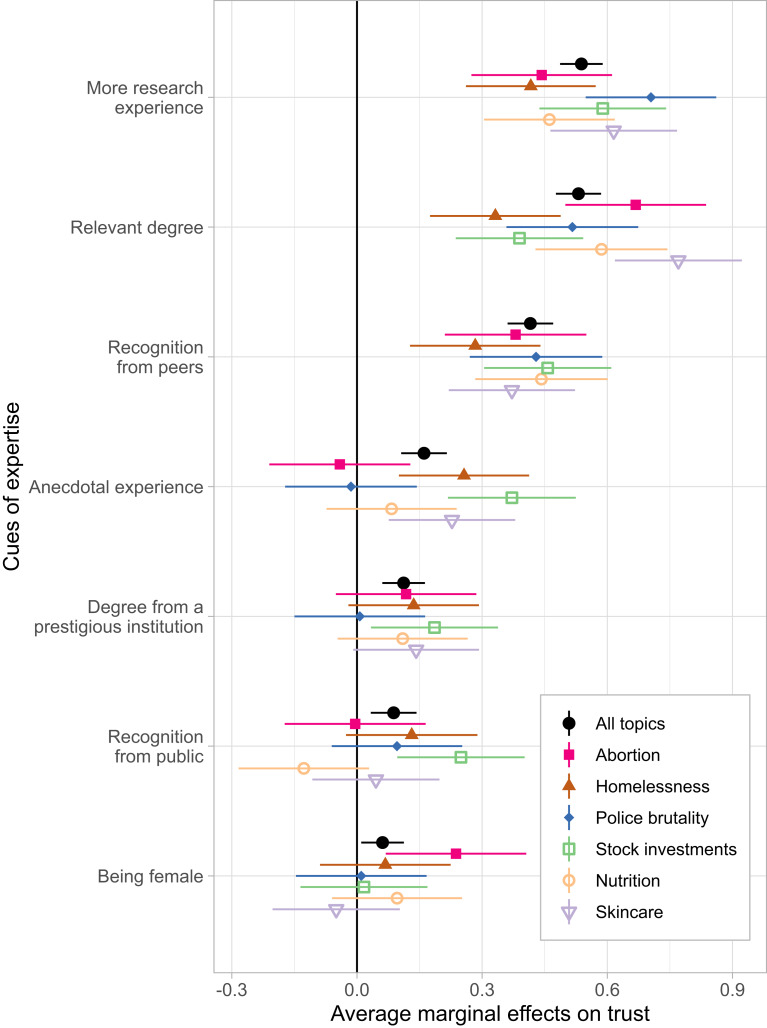
Effects of expertise cues on trust (Study 2). Estimates are standardized. Lines represent 95% confidence intervals.

We found that people trusted an expert more when the expert had researched the topic for a longer time, had a degree that was relevant to the topic, or received more recognition from the scientific community (Fig. 2). Those three attributes, which we would ordinarily think of as good cues of an expert’s knowledge, had the largest impact on trust across all topics (an increase of 0.54, 0.53, and 0.41 SDs, respectively). But we also found that other cues that are weaker signals of knowledge nevertheless influenced trust. People trusted an expert more if they had some anecdotal experience on the topic, a degree from an Ivy League institution, or many followers of social media. The impact of these poor cues was smaller and less consistent across topics (an increase of 0.16, 0.11, 0.09 SDs, respectively).

Aside from trust, we also measured people’s impressions of the expert’s specific attributes (e.g., How much time did they spend studying the topic? How much recognition do they receive from other experts? How relevant is their academic background?). People trusted an expert more to the extent they *inferred* that the expert spent a lot of time studying the topic, received recognition from their peers, and so on (Supplementary Table S1). Even though the attributes of experts were independently manipulated, people’s impressions of these attributes had small to moderate correlations among them. Furthermore, we observed a “spillover” effect, such that manipulating a single attribute impacted impressions of multiple attributes, in addition to the one that the manipulation targeted. For example, when an expert had an undergraduate major that was relevant to the topic, people also thought the expert had studied the topic longer, had graduated from a more prestigious institution, received more public recognition, and so on, regardless of the information they received about the latter attributes (Supplementary Table S2).

As with the first study, we observed context-specific effects for attributes such as gender (Fig. 2). People thought that a female expert had more personal experience on the topic of abortion and trusted her more. Replicating the importance placed on the expert’s wealth for stock investments, we found that for a stock expert, being a self-made multimillionaire was equivalent to having a degree in Finance (vs. Classics). Taken together, the first two studies revealed considerable overlap in what people *said* mattered and what *actually* mattered for their judgments, at least when they didn’t know the expert’s view on an issue.

### Study 3

The first two studies showed that when people lacked information about an expert’s views, they trusted an expert with relevant academic backgrounds and more experience. We wanted to test whether or not people would still recognize those cues when the expert shared their strong, moralized opinions. We chose a moralized issue where people’s opinions split into two camps: abortion. Participants (*n =* 1,776; 940 pro-choice, 862 pro-life) read about a researcher who had written a best-selling book on abortion, who either had a medical degree and had spent a long time researching and publishing *or* one who had a mechanical engineering degree and had less research experience. In addition to the level of expertise, we manipulated the researcher’s views (i.e., pro-life vs. pro-choice vs. no information; we coded the first two as congruent vs. incongruent based on participants’ views) and gender. As pre-registered, we used the aggregated trust items (*α* = 0.96) as our main outcome and also measured impressions of specific attributes, similar to Study 2 (pre-registration for this experiment can be found here: https://osf.io/j6v4f/).

**Fig. 3:**
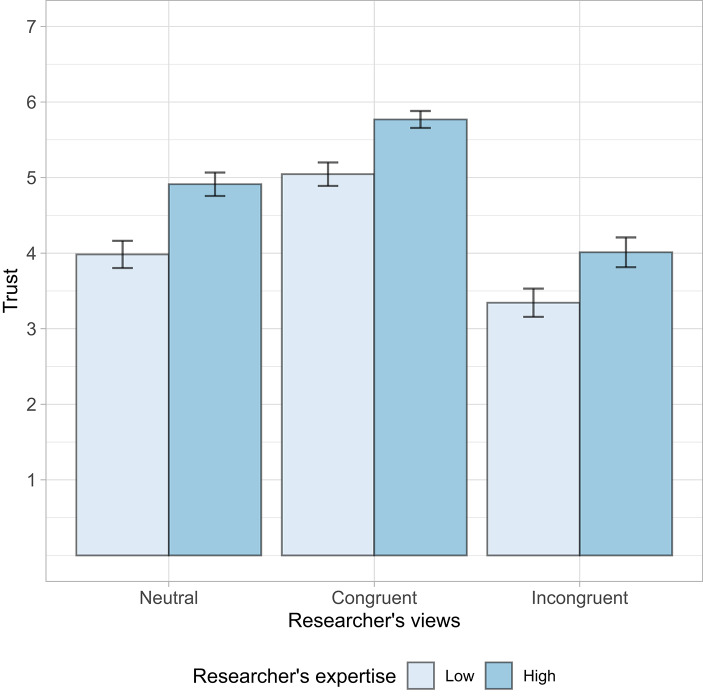
Effects of the researcher’s expertise and views on trust (Study 3). Error bars represent 95% confidence intervals.

Overall, participants trusted the qualified researcher more than the less-qualified one (Fig. 3). They also trusted the researcher who shared their political views more than the one who did not. The effect of the researcher’s political views (*η*^*2*^_*G*_ = 0.195) was more than twice as large as the effect of expertise (*η*^*2*^_*G*_ = 0.075), and the two did not interact (Supplementary Table S3; see Supplementary Table S4 for results broken down for each trust item). The magnitude of the effect was striking given that participants in the first study didn’t rate an expert’s political preferences as highly relevant. Participants trusted a less-qualified researcher who shared their views (*M* = 5.05, *SD* = 1.36) just as much as they trusted a highly-qualified researcher whose views they didn’t know (*M* = 4.92, *SD* = 1.31). Similarly, participants trusted a highly-qualified researcher who held different views (*M* = 4.01, *SD* = 1.68) only as much as a less-qualified researcher whose views they didn’t know (*M* = 3.98, *SD* = 1.57). We found no evidence that pro-life and pro-choice participants differed in the strength of bias toward researchers who shared their views (Participant views x Researcher views interaction: *F*(2, 1759) = 1.72, *p* =.179), or in how well they recognized credentials (Participant views x Expertise interaction: *F*(1, 1759) = 0.93, *p* =.336; Supplementary Table S5). In line with our previous studies, people thought that a female researcher had more personal experience on abortion (*F*(1, 1763) = 4.73, *p* =.03). That did not translate into more trust in her across the board (*F*(1, 1759) = 3.45, *p* =.063). Instead, we found that only pro-choice participants trusted the female researcher slightly more, whereas we did not observe a gender preference among pro-life participants (Participant views x Gender interaction: *F*(1, 1759) = 12.23, *p* <.001; Supplementary Figure S1).

**Fig. 4:**
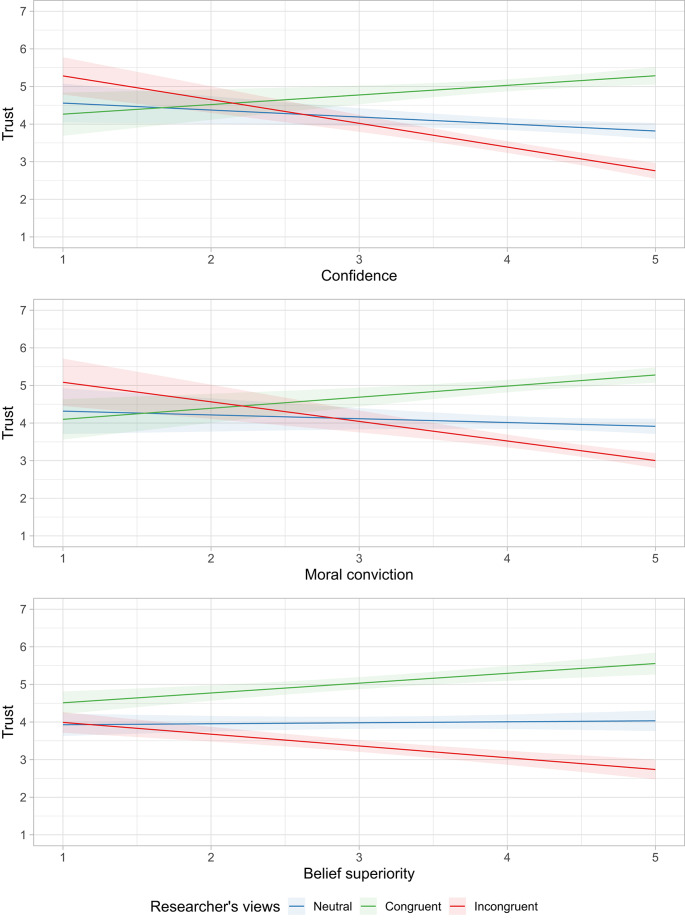
Individual differences in confidence, moral conviction, and belief superiority moderating the effect of the researcher’s views on trust (Study 3).

As expected, the effect of the researcher’s views on participant trust was larger among those who were more invested in their beliefs compared to those who weren’t as invested (Fig. 4). In other words, participants who had stronger moral convictions, confidence, and belief superiority made sharper distinctions between a researcher who shared their views compared to one who did not. Contrary to our expectations, being more invested in one’s beliefs did not interact with the effect of expertise when the researcher’s views were known (Supplementary Table S6). Participants distinguished between a qualified vs. unqualified researcher regardless of how much participants were invested in their beliefs.

We also replicated the “spillover” effect from Study 2 (Supplementary Table S7). Even though our expertise manipulation only entailed differences in years of experience and relevance of academic background, the manipulation also affected impressions of other aspects of expertise that were either not mentioned (e.g., personal experience) or fixed between conditions (e.g., prestige of academic institution) (*p*s < 0.001). Interestingly, we found that the researcher’s views also influenced all of the participants’ impressions (*p*s < 0.001). In other words, when people noticed that a researcher shared their views, they thought the researcher had spent more time studying the topic, had a more relevant academic background, and so on. We should note that the effect of the researcher’s views on specific impressions (0.014 < *η*^*2*^_*G*_’s < 0.050; Supplementary Table S7) was much smaller than its effect on trust.

Finally, we asked participants to guess the researcher’s views in the condition where those views were not explicitly stated. Our prediction was that participants who were more invested in their beliefs would assume that the researcher shared their views, especially when the researcher was highly qualified. However, that was not the case (Supplementary Table S8). We also tested whether participants made guesses about the researcher’s views based on stereotypes. For example, participants might have assumed that the highly-qualified researcher would be pro-choice because educated people tend to be more liberal or that a male researcher would be pro-life. The researcher’s expertise or gender did not seem to influence people’s guesses, however (Supplementary Table S9). That suggests that people trusted good cues of expertise for their own sake, not because those cues allowed them to make assumptions about the researcher’s views.

## Discussion

Our results reveal a complicated picture of laypeople’s evaluations of expertise. On the one hand, we found evidence that people are generally attentive to good cues of expertise (e.g., relevant training and experience) whereas they are less attentive to poor cues (e.g., ethnicity and height). We observed some variability in how much participants weighed those factors in their evaluations of experts across topics, but good cues were generally given more weight than poor ones, at least when the expert’s views were not known. That was true in both participants’ self-reports (Study 1) and their reactions to manipulations of various cues in a conjoint experiment (Study 2).

On the other hand, Study 3 revealed clear evidence of partisan biases in evaluating experts. When we specified an expert’s views on abortion, participants were significantly influenced by the expert’s opinions when determining the expert’s trustworthiness and credibility. While they remained attentive to good cues, the effect of congeniality with the expert’s expressed views was over twice as large as the effect of an expert’s having a relevant academic background; the discrepancy between the two effects was the greatest for perceptions of trustworthiness (Supplementary Table S4). In fact, participants trusted experts who agreed with them but had an irrelevant academic background (mechanical engineering) and limited experience significantly more than experts who disagreed with them but had a more relevant background (medicine) and substantially more experience. These results suggest a more pessimistic view of laypeople’s evaluations of experts on topics that are moralized and politicized.

The large effect size we observed for the expert’s views in Study 3 stands in contrast with the low self-reported relevance of experts’ politics in Study 1. It could be that people are unaware that their judgments can be affected by poor cues (including superficial characteristics like physical appearance) in the first place^38^. Alternatively, people may consciously prefer likeminded experts, but be motivated to understate how much they value party allegiances over other good cues, in an effort to appear unbiased^39,40^We did not explicitly ask participants to describe their thought process in Study 3, but it’s also possible that, when questioned, they might launder their partisan preferences through more acceptable cues they reported as relevant in Study 1, like moral character (e.g., “I trust the likeminded expert because they are less likely to deceive me.”). Some might acknowledge their partisan preferences more readily. Those who had stronger moral convictions, for example, not only showed more partisan bias (Study 3) but also appraised an experts’ politics as more relevant for expertise (Study 1) —in line with recent evidence showing that people are comfortable admitting to being biased for moral ends^41^. Future research should examine how people justify their preferences for likeminded experts, and whether there are lay beliefs about the nature of expertise that can be corrected by interventions^42^.

People’s impressions of specific cues of expertise were similarly complicated. We found that judgments about the relevance of an expert’s academic background or how much they are recognized by other experts largely tracked actual cues that we manipulated. But these judgments were also influenced by irrelevant cues. Some of this “spillover” can be explained by people making judgments beyond the information they have, based on reasonable assumptions. For example, when people see an expert who has published many articles and received many awards, they might assume that the expert has probably also received public recognition. But it is less reasonable to think that a mechanical engineer’s academic background should become more pertinent to abortion when the engineer agrees with one’s views, something which we observed in Study 3. It is likely that people initially formed a holistic impression of the expert and that impression then influenced more specific judgments—akin to the halo effect^43,44^—thus allowing people to rationalize their preference for likeminded experts (e.g., “Why should I trust this expert who thinks like I do? Because they have a relevant academic background.”). Our findings show that while lay judgments of expertise might generally track normative standards of rationality, they can also be influenced by myside biases and coalitional motivations^20,21,45^. While these biases can serve social functions, such as signaling group commitment, they can trap us in a closed cognitive loop, seeking experts who tell us what we already believe or want to believe. Future work should investigate how to decouple assessments of experts’ qualities from whether or not they agree with us.

We must acknowledge that our selection of stimulus materials might limit generalizability. We aimed to maximize the effect sizes when we manipulated credentials. But the differences between experts and non-experts might be less stark in real life. Although laypeople might find a mechanical engineer unqualified to offer medical advice, a chemist (as in the example of Linus Pauling) might be deemed “close enough” to weigh in. When we wanted to manipulate the expert’s political views, we picked a topic where most people already held opinions and observed a rather large effect. But for other topics—unlike abortion—that haven’t been the focus of intense policy debates and culture wars, expertise might prevail over politics.

Our results add important nuance to discussions of the “crisis of expertise.” While our participants were generally more attuned to good cues than the most pessimistic commenters might have predicted, we also found that our participants were prone to congeniality biases. These biases gave people inflated trust in ideologically “friendly” experts and deflated trust in ideologically “unfriendly” ones, independently of the experts’ actual credentials. Sometimes, people roughly know what qualities to look for in experts, but because they’re aware of how a suitable expert should look, they might invent or overemphasize a friendly expert’s qualities in order to rationalize their trust in that expert. Our findings thus reflect a social demand for *congenial expertise*, rather than a general, across-the-board distrust in expertise. In situations where no legitimate experts are willing or able to defend policy positions put forward by politicians (e.g., opposition to all vaccine mandates), laypeople who support those positions turn to “influencers” as sources of news and scientific information^30,46^. Imagine for a moment that all of our current policy debates hinged on issues where there was genuine scientific disagreement and thus a lack of robust consensus. In that case, it’s easy to imagine there would be an adequate supply of ideologically friendly experts for all partisans. But even here the public would still be divided about facts, though without any “crisis of expertise” in some popular senses of the term. The current crisis, then, is not an outright dismissal of the value of expertise. Instead, it appears to be a crisis of selectively elevating or denigrating available experts in order to make one’s preferred views appear more justified. The old wisdom that “authority has a nose of wax” finds support in our data. And when everybody believes they are following authoritative experts, broad appeals to expert authority (e.g., “trust the science”) might fall flat.

Our findings do not reveal any readymade solutions for improving laypeople’s evaluations of expert cues in contexts of political disagreement and polarization. Indeed, we found that participant agreement with an expert’s opinion was twice as strong a predictor of participant trust than the expert having a relevant academic background. Unfortunately, that pattern of trust makes the public vulnerable to misbehaving or cynical experts. Our evidence suggests that epistemic trespassers^24^might have social advantages over legitimate experts when they publicly express opinions demanded by an audience. While there might not be an epistemically ideal way to satisfy partisans’ demand for congenial expertise, our findings at least suggest that holding epistemic trespassers to account might help to improve partisans’ information supply. Due to the powerful social and economic incentives that some experts have for transforming into geniuses and oracles on the public stage^47^, preventing them from trespassing is itself an ambitious goal. The best hope for an epistemic community might be to better legislate its boundaries and help direct laypeople toward the groups of experts who have the most legitimate claims to expertise.

## Methods

All studies were carried out in accordance with the policies and procedures of the Human Research Protection Program (HRPP) as well as the ethical guidelines of the Human Research Protections unit of the Office of Research at the University of California - Irvine. Study protocols were considered exempt from review (Categories 2 and 3) by the Institutional Review Board at the University of California - Irvine (protocol #5756). All participants gave informed consent and received compensation.

### Study 1

The study was conducted on Oct 7, 2024. We recruited a gender-balanced sample of 210 U.S. adults from Prolific, stratified by ideology to cover the entire political spectrum (i.e., 70 liberals, 70 moderates, 70 conservatives). After filtering out 2 participants who failed the attention check question, the final sample consisted of 208 participants.

#### Materials and procedure

Participants were asked to imagine a scenario where they were trying to gather information on a topic and figure out who is an expert on that topic. They saw a random subset of 3 out of 6 topics: skin products, nutrition, stock investments, abortion, homelessness, and police brutality.

For each topic, participants were presented a list of 18 attributes that a potential expert might have. The order of these attributes were randomized across participants but kept fixed within participants. Participants reported how relevant each attribute would be when figuring out who is an expert on that topic using a 5-point scale from “Not relevant at all” to “Extremely relevant”. The list of attributes was as follows:

**Table:** 

● Years of education they had	● Their confidence
● Whether they have a degree related to that specific topic	● The party they vote for
● Whether they have personal experience regarding that topic	● Their height
● Their level of intelligence in general	● Their gender
● Where they work	● Their age
● Financial incentives they might have	● Their race
● The recognition they received from their peers (e.g., awards, prizes)	● Their looks
● Their reputation among the general public	● Their sexual orientation
● Their moral character	● Their wealth

Following the relevance judgments for each topic, participants were presented with three items about the topic in question, designed to assess strength of prior beliefs, strength of moral conviction, and perceived politicization. Participants reported their agreement on a 5-point scale.

- I have strong opinions about [topic].
- My feelings about [topic] are a reflection of my core moral beliefs and convictions.
- The topic of [topic] is politicized.

After the block of questions about attributes and topic, participants answered an attention check question. Finally, they reported their demographics (i.e., age, gender, race, household income, education), as well as their political orientation (i.e., liberal vs. conservative), party identification, and interest in politics.

#### Data analysis

For the analyses regarding relevance of the expert’s political preferences, we used a multilevel model that included random intercepts for participants and topics, with strength of moral convictions, perceived politicization, ideology, interaction between moral convictions x ideology, and interaction between perceived politicization and ideology as predictors. Ideology was centered around moderates, whereas the outcome variable and the remaining predictors were standardized. To test the context-specific effects of certain attributes (i.e., wealth, gender, looks) we used multilevel models that included random intercepts for participants, with topic as the predictor; we reported the omnibus effect of topic (calculated using the Anova() function from the *car* package in R^48^) in the main text.

### Study 2

The study was fielded between December 16–19, 2024. We recruited a sample of 501 adult participants from Prolific that meant to represent the demographics of the U.S. population. After filtering out 3 participants who failed the attention check question, the final sample consisted of 498 participants.

#### Materials and procedure

Participants read short biographies of fictional experts on all of the aforementioned 6 topics and reported their trust toward the experts. In each biography, seven attributes of the expert were simultaneously manipulated, following a conjoint design. With two levels for each factor, each participant was only exposed to a small fraction of all possible combinations (2^7^). However, for each factor, half of the 6 observations from each participant was randomly assigned to one level or another. The list of factors and their levels were as follows (please refer to the Supplementary Material for the exact stimuli):

- Gender (male vs. female).
- Years of experience (high vs. low).
- Relevance of degree (high vs. low).
- Prestige of institution (high vs. low).
- Recognition from peers (present vs. absent).
- Recognition from the public (present vs. absent).
- Anecdotal experience (present vs. absent).

Based on the biography, participants responded to two groups of items about the expert. The first one consisted of measures about the expert’s perceived expertise, and participants’ trust in them. Participants reported their agreement on a 7-point scale. Reliability scores ranged from 0.95 to 0.97 across topics:

- This person is an expert on [topic].
- This person knows what they are talking about when it comes to [topic].
- I would trust this person’s opinion on [topic].
- This person is a credible source of information on [topic].

The second group of questions measured impressions of specific attributes of the expert, each one targeted by an experimental manipulation. Participants answered these questions on a 5-point scale from “None at all” to “A great deal”. The questions and the corresponding experimental manipulations (in parentheses) were as follows:

- How much time did they spend studying [topic]? (Years of experience)
- How relevant is their academic background to [topic]? (Relevance of degree)
- How prestigious is the academic institution where they studied? (Prestige of institution)
- How much recognition do they receive from experts working on [topic]? (Recognition from peers)
- “How much recognition do they receive from the public? (Recognition from the public)
- How much personal experience do they have when it comes to [topic]? (Anecdotal experience)

To check for potential order effects, each participant was assigned to see either the questions on trust or the questions on specific attributes first. Similar to the first study, the main question block was followed by an attention check, and questions on demographics and politics.

#### Data analysis

For the analyses with trust as the outcome variable, we averaged the four items measuring trust toward the expert and used the composite score. To test the effects of experimental manipulations on judgments of trust and impressions of specific attributes, we used multilevel models with random intercepts for participants and topics; the models conducted on subsets of data split up by topic only had random intercepts for participants. We conducted conjoint analyses where we calculated the average marginal component effect (AMCE) for each manipulation using the *marginaleffects* R package^49^.

### Study 3

The study was conducted on May 10, 2025. We recruited a sample of 1803 adult participants from Prolific. We aimed to recruit equal numbers of pro-life and pro-choice participants by pre-screening them based on the views of abortion they reported when they signed up to the platform. After filtering out 27 participants who failed the attention check question, the final sample consisted of 1,776 participants.

#### Materials and procedure

Participants started the survey by reporting their views on abortion (Pro-choice vs. Pro-life) which was used to validate Prolific’s pre-screener. After the validation, they were asked three follow-up questions to measure participants’ level of investment in their views on abortion:

- Confidence: “How confident are you in your views on abortion?” (5-point scale from”Not confident at all” to “Extremely confident”).
- Belief superiority (from Toner et al.^50^): “In your view, how much more correct are your beliefs about abortion than other people’s beliefs about this issue?” (5-point scale with the following options: “No more correct than other viewpoints”, “Slightly more correct than other viewpoints”, “Somewhat more correct than other viewpoints”, “Much more correct than other viewpoints”, “Totally correct (Mine is the only correct view)”).
- Moral conviction: “My feelings about abortion are a reflection of my core moral beliefs and convictions.” (5-point Agree-Disagree scale).

After participants answered the questions about their own beliefs, they read the biography of a fictional researcher who wrote a best-selling book. The biography resembled a Wikipedia page in style and appearance. It had a short intro section containing the researcher’s age and their best-selling book, followed by a “Education and career” section. Three factors about the researcher were manipulated in a between-subjects fashion: the researcher’s expertise (high vs. low), their views (pro-choice vs. pro-life vs. no information), and their gender (male vs. female).

To manipulate expertise, we combined two of the expertise cues that had the strongest effects in Study 2: relevance of major and years of experience. In the high expertise condition, the expert had a major that was relevant to the topic (Medicine), started publishing and researching right after graduating, and wrote two books before his best-seller. In the low expertise condition, the expert had a major that was not relevant to the topic (Mechanical Engineering), worked for 10 years at a consulting company, and only started researching the topic 3 years before the best-seller. This information was presented under the “Education and career” section. To manipulate views, we changed the section of the biography that followed “Education and career”. For the pro-choice and pro-life conditions, participants read a section titled “Views” that described the researcher’s views and how they wrote about the evidence supporting those views. In the No-information condition, participants read a section titled “Personal life” about their residence and their spouse. To manipulate gender, we changed the researcher’s name: “Daniel” for the male expert and “Sophia” for the female expert.

Based on the biography, participants responded to two groups of measures about the expert, similar to Study 2. They first responded to the items related to how much they trust the expert (*α* = 0.96), followed up by their impressions of the expert’s attributes (e.g., how long they spent studying, how relevant their background is, etc.). They also answered an attention check question among the second group of items.

After the main task, participants were asked two comprehension check questions. One was about the expert’s major, the other one was about the expert’s views (Pro-choice vs. Pro-life vs. Don’t know). Finally, they answered questions on demographics and politics.

#### Data analysis

We coded the pro-life and pro-choice conditions as Congruent or Incongruent depending on the participants’ views on abortion. To test how the experimental manipulations affected trust, we used a 2 [Expertise: Low vs. High] x 3 [Views: Congruent vs. Incongruent vs. No-information] x 2 [Researcher Gender: Male vs. Female] factorial ANOVA with the composite score for the four trust items as the dependent variable. To test whether the effects differ between pro-life vs. pro-choice participants, we used a separate model that included the participants’ views on abortion (pro-choice vs. pro-life), as well as its two-way interactions with all three factors. To test the “spillover effect”, we used separate ANOVA models with the three main factors, with impressions of specific attributes as the dependent variables.

To test the effect of being invested in one’s beliefs on trust, we used three separate linear regression models for each of the following individual-difference variables: confidence, belief superiority, and moral conviction. The models included the researcher’s expertise, the researcher’s views, the individual difference variable (standardized), and their 2- and 3-way interactions as predictors, and the trust composite as the outcome. As pre-registered, we treated these models as being in the same “family”, and adjusted the p-values using the Holm correction to keep the error rate in check.

To examine people’s guesses about the expert’s views when there is no explicit information, we used the subset of our data that only contains the responses from the no-information condition. We ran a multinomial regression model with people’s guesses about the researcher’s views (i.e., pro-choice or pro-life) as outcomes, and “Don’t know” as the reference category. We included researcher’s expertise and gender as predictors.

To test whether people who are invested in their beliefs project their views onto the researchers, we first coded the guesses about the expert’s views as congruent or incongruent with the participant’s views; then we created a binary variable that indicates projection of one’s own views onto the expert (i.e., 1 = congruent, 0 = incongruent/Don’t know). We used this binary variable as the outcome for three separate logistic regression models for each individual difference variable, with expertise, the individual difference variable, and their interaction as predictors.

## Supplementary Information

Below is the link to the electronic supplementary material.

Supplementary Material 1

## Data Availability

The datasets generated for the current studies, along with the analysis scripts, are available on Open Science Framework: https://osf.io/nxmkc/.

## References

[1] Ballantyne, N., Celniker, J. B., Dunning, D. Do your own research. *Soc. Epistemol*. **38**, 302–317 (2024).

[2] Chinn, S. & Hasell, A. Support for doing your own research is associated with COVID-19 misperceptions and scientific mistrust. *Harv. Kennedy Sch. Misinformation Rev.*10.37016/mr-2020-117 (2023).

[3] Drezner, D. *The Ideas Industry: How Pessimists, Partisans, and Plutocrats Are Transforming the Marketplace of Ideas* (Oxford University Press, 2017).

[4] Chinn, S., Hiaeshutter-Rice, D. & Chen, K. How science influencers polarize supportive and skeptical communities around politicized science: A Cross-Platform and Over-Time comparison. *Polit Commun.* **41**, 627–648 (2024).

[5] Eyal, G. Mistrust in numbers: Regulatory Science, trans-science and the crisis of expertise. *Spontaneous Gener J. Hist. Philos. Sci.* **10**, 36–46 (2022).

[6] Oreskes, N. & Conway, E. M. *Merchants of Doubt: How a Handful of Scientists Obscured the Truth on Issues from Tobacco Smoke To Climate Change* (Bloomsbury Publishing, 2022).

[7] Nichols, T. M. *The Death of Expertise: The Campaign Against Established Knowledge and Why It Matters*. (Oxford University Press, New York, NY, 2017).

[8] Kitcher, P. *The Advancement of Science: Science Without Legend, Objectivity Without Illusions* (Oxford University Press, 1995).

[9] Goldman, A. I. Experts: Which ones should you trust? *Philos. Phenomenol Res.* **63**, 85–110 (2001).

[10] Hardwig, J. Epistemic dependence *J. Philos.* **82**, 335–349 (1985).

[11] Hardwig, J. The role of trust in knowledge. *J. Philos.* **88**, 693–708 (1991).

[12] Ballantyne, N. Novices and expert disagreement. In *Reason, Bias, and Inquiry* (eds. Ballantyne, N. & Dunning, D.) 227–253. 10.1093/oso/9780197636916.003.0011 (Oxford University Press, 2022).

[13] Anderson, E. Democracy, public policy, and lay assessments of scientific testimony. *Episteme* **8**, 144–164 (2011).

[14] Croce, M. & Baghramian, M. Experts – Part I: what they are and how to identify them. *Philos. Compass*. **19**, e13009 (2024).

[15] Keil, F. C., Stein, C., Webb, L., Billings, V. D. & Rozenblit, L. Discerning the division of cognitive labor: an emerging understanding of how knowledge is clustered in other Minds. *Cogn. Sci.* **32**, 259–300 (2008). 19759842 10.1080/03640210701863339 PMC2744112

[16] Lutz, D. J. & Keil, F. C. Early understanding of the division of cognitive labor. *Child. Dev.* **73**, 1073–1084 (2002). 12146734 10.1111/1467-8624.00458

[17] Golbeck, J. & Fleischmann, K. R. Trust in social Q&A: the impact of text and photo cues of expertise. *Proc. Am. Soc. Inf. Sci. Technol.* **47**, 1–10 (2010).

[18] Harvey, N., Fischer, I. Taking advice: Accepting help, improving judgment, and sharing responsibility. *Organ. Behav. Hum. Decis. Process.* **70**, 117–133 (1997).

[19] Gugerty, L. & Link, D. M. How heuristic credibility cues affect credibility judgments and decisions. *J. Exp. Psychol. Appl.* **26**, 620–645 (2020). 32658526 10.1037/xap0000279

[20] Mercier, H. *Not Born Yesterday: The Science of Who We Trust and What We Believe* (Princeton University Press, Princeton, NJ, 2020).

[21] Sperber, D. et al. Epistemic vigilance. *Mind Lang.* **25**, 359–393 (2010).

[22] Eom, D. et al. Race and gender biases persist in public perceptions of scientists’ credibility. *Sci. Rep.* **15**, 11021 (2025). 40164677 10.1038/s41598-025-87321-z PMC11958661

[23] Taleb, N. N. *Fooled by Randomness: the Hidden Role of Chance in Life and in the Markets* (Random House Trade Paperbacks, 2005).

[24] Ballantyne, N. Epistemic trespassing. *Mind* **128**, 367–395 (2019).

[25] Ditto, P. H., Celniker, J. B., Siddiqi, S. S., Güngör, M. & Relihan, D. P. Partisan bias in political judgment. *Annu. Rev. Psychol.* **76**, 717–740 (2025). 39237099 10.1146/annurev-psych-030424-122723

[26] Schaffner, B. F. & Luks, S. Misinformation or expressive responding? What an inauguration crowd can tell us about the source of political misinformation in surveys. *Public. Opin. Q.* **82**, 135–147 (2018).

[27] Yair, O. & Huber, G. A. How robust is evidence of partisan perceptual bias in survey responses? A new approach for studying expressive responding. *Public. Opin. Q.* **84**, 469–492 (2020).

[28] Bullock, J. G., Gerber, A. S., Hill, S. J. & Huber, G. A. Partisan bias in factual beliefs about politics. *Q. J. Polit Sci.* **10**, 519–578 (2015).

[29] Wallace, J., Goldsmith-Pinkham, P. & Schwartz, J. L. Excess death rates for Republican and Democratic registered voters in Florida and Ohio during the COVID-19 pandemic. *JAMA Intern. Med.* **183**, 916 (2023). 37486680 10.1001/jamainternmed.2023.1154 PMC10366951

[30] Newman, N., Arguedas, R., Robertson, A., Nielsen, C. T. & Fletcher, R. R. K. *Reuters Insitute Digital News Report 2025*. https://reutersinstitute.politics.ox.ac.uk/digital-news-report/2025 (2025). 10.60625/RISJ-8QQF-JT36

[31] Celniker, J. B. & Ditto, P. H. Of preferences and priors: Motivated reasoning in partisans’ evaluations of scientific evidence. *J. Pers. Soc. Psychol.* **127**, 986–1011 (2024). 39636596 10.1037/pspa0000417

[32] Ditto, P. H. et al. At least bias is bipartisan: A meta-analytic comparison of partisan bias in liberals and conservatives. *Perspect. Psychol. Sci.* **14**, 273–291 (2019). 29851554 10.1177/1745691617746796

[33] Washburn, A. N. & Skitka, L. J. Science denial across the political divide: Liberals and conservatives are similarly motivated to deny attitude-inconsistent science. *Soc. Psychol. Personal Sci.* **9**, 972–980 (2018).

[34] Bansak, K. et al. Conjoint survey experiments. *Adv Exp. Polit Sci* **19**, (2021).

[35] Hainmueller, J., Hopkins, D. J. & Yamamoto, T. Causal inference in conjoint analysis: Understanding multidimensional choices via stated preference experiments. *Polit Anal.* **22**, 1–30 (2014).

[36] Hovland, C., Janis, I., & Kelley, H.* Communication and Persuasion: Psychological Studies of Opinion Change* (Yale University Press, New Haven, CT, 1976).

[37] Pornpitakpan, C. The persuasiveness of source credibility: A critical review of five decades’ evidence. *J. Appl. Soc. Psychol.* **34**, 243–281 (2004).

[38] Nisbett, R. E. & Wilson, T. D. Telling more than we can know: verbal reports on mental processes. *Psychol. Rev.* **84**, 231–259 (1977).

[39] Cruz, F. & Mata, A. Motivated bias blind spot: People confess to more or less bias depending on its desirability. *Mind Soc.* **24**, 341–358 (2025).

[40] Pronin, E. Perception and misperception of bias in human judgment. *Trends Cogn. Sci.* **11**, 37–43 (2007). 17129749 10.1016/j.tics.2006.11.001

[41] Cusimano, C. & Lombrozo, T. People recognize and condone their own morally motivated reasoning. *Cognition* **234**, 105379 (2023). 36791606 10.1016/j.cognition.2023.105379

[42] Cusimano, C. & Lombrozo, T. Reconciling scientific and commonsense values to improve reasoning. *Trends Cogn. Sci.* **25**, 937–949 (2021). 34281766 10.1016/j.tics.2021.06.004

[43] Nisbett, R. E. & Wilson, T. D. The halo effect: Evidence for unconscious alteration of judgments. *J. Pers. Soc. Psychol.* **35**, 250–256 (1977).

[44] Wetzel, C. G., Wilson, T. D. & Kort, J. The halo effect revisited: Forewarned is not forearmed. *J. Exp. Soc. Psychol.* **17**, 427–439 (1981).

[45] Barlev, M. & Neuberg, S. L. Rational reasons for irrational beliefs. *Am. Psychol.* **80**, 79–90 (2025). 38619485 10.1037/amp0001321

[46] O’Brien, G., Ganjigunta, R. & Dhillon, P. S. Wellness influencer responses to COVID-19 vaccines on social media: A longitudinal observational study. *J. Med. Internet Res.* **26**, e56651 (2024). 10.2196/56651 PMC11635329 39602782

[47] Fahy, D. *The New Celebrity Scientists: Out of the Lab and into the Limelight* (Rowman & Littlefield, Lanham, MD, 2015).

[48] Fox, J. et al. car: Companion to Applied Regression. (2024).

[49] Arel-Bundock, V., Greifer, N. & Heiss, A. How to interpret statistical models using marginaleffects for R and python. *J. Stat. Softw.* **111**, 1–32 (2024).

[50] Toner, K., Leary, M. R., Asher, M. W. & Jongman-Sereno, K. P. Feeling superior is a bipartisan issue: Extremity (not direction) of political views predicts perceived belief superiority. *Psychol. Sci.* **24**, 2454–2462 (2013). 24096379 10.1177/0956797613494848

