# Supplementary material for: Politics matter more than credentials in laypeople’s judgments of expertise
Source: Sci Rep. 2026 Mar 9;16:12765. doi: 10.1038/s41598-026-40053-0 (PMC13096159; doi:10.1038/s41598-026-40053-0)
Supplement: Supplementary file 1 — Supplementary Material 1 [file 41598_2026_40053_MOESM1_ESM.docx]

Supplementary materials

For “Politics matter more than credentials in laypeople’s judgments of expertise”

Contents

[Sample characteristics 2](#_Toc203947143)

[Study 1 2](#_Toc203947144)

[Study 2 3](#_Toc203947145)

[Study 3 4](#_Toc203947146)

[Stimulus materials 7](#_Toc203947147)

[Study 1 7](#_Toc203947148)

[Relevance questions: 7](#_Toc203947149)

[Follow-up questions about the topics: 7](#_Toc203947150)

[Wordings for the topics 8](#_Toc203947151)

[Study 2 9](#_Toc203947152)

[Expert biographies 9](#_Toc203947153)

[Trust questions 11](#_Toc203947154)

[Impressions of specific attributes 11](#_Toc203947155)

[Study 3 13](#_Toc203947156)

[Screening question 13](#_Toc203947157)

[Follow-up questions 13](#_Toc203947158)

[Biographies 14](#_Toc203947159)

[Comprehension checks 26](#_Toc203947160)

[Supplementary tables and figures 27](#_Toc203947161)

[Figure S1 27](#_Toc203947162)

[Table S1 28](#_Toc203947163)

[Table S2 29](#_Toc203947164)

[Table S3 30](#_Toc203947165)

[Table S4 31](#_Toc203947166)

[Table S5 32](#_Toc203947167)

[Table S6 33](#_Toc203947168)

[Table S7 34](#_Toc203947169)

[Table S8 35](#_Toc203947170)

# Sample characteristics

## Study 1

| **Characteristic** | **N = 208** |
| --- | --- |
| Age | 39.0 (12.0) |
| Gender |  |
| Female | 104 (50%) |
| Male | 102 (49%) |
| Non-binary | 2 (1.0%) |
| Race/Ethnicity |  |
| White | 157 (75%) |
| Black | 20 (9.6%) |
| Latino | 20 (9.6%) |
| Asian | 23 (11%) |
| Party |  |
| Democrat | 93 (45%) |
| Independent | 38 (18%) |
| Republican | 77 (37%) |
| Ideology |  |
| Conservative | 75 (36%) |
| Liberal | 92 (44%) |
| Moderate | 41 (20%) |

## Study 2

| **Characteristic** | **N = 498** |
| --- | --- |
| Age | 45.7 (16.2) |
| Gender |  |
| Female | 248 (50%) |
| Male | 243 (49%) |
| Non-binary | 6 (1.2%) |
| Other | 1 (0.2%) |
| Race/Ethnicity |  |
| White | 352 (71%) |
| Black | 75 (15%) |
| Latino | 44 (8.9%) |
| Asian | 53 (11%) |
| Party |  |
| Democrat | 203 (41%) |
| Independent | 125 (25%) |
| Republican | 170 (34%) |
| Ideology |  |
| Conservative | 178 (36%) |
| Liberal | 213 (43%) |
| Moderate | 107 (21%) |

## Study 3

| **Characteristic** | **N = 1,776** |
| --- | --- |
| Age | 42.7 (14.0) |
| Gender |  |
| Female | 877 (49%) |
| Male | 885 (50%) |
| Non-binary | 11 (0.6%) |
| Other | 3 (0.2%) |
| Race/Ethnicity |  |
| White | 1,289 (73%) |
| Black | 355 (20%) |
| Latino | 106 (6.0%) |
| Asian | 80 (4.5%) |
| Party |  |
| Democrat | 668 (38%) |
| Independent | 263 (15%) |
| Republican | 845 (48%) |
| Ideology |  |
| Conservative | 834 (47%) |
| Liberal | 685 (39%) |
| Moderate | 257 (14%) |
| Views on abortion |  |
| Pro-choice | 940 (53%) |
| Pro-life | 836 (47%) |

#

# Stimulus materials

## Study 1

### Relevance questions:

“Imagine you are trying to gather information on [topic]. You want to figure out who to trust on that topic. You will see a list of attributes below. How relevant do you think each of these attributes are when figuring out who is an expert on that topic?”

Participants rated the relevance of the following attributes on a 5-point scale from “Not relevant at all” to “Extremely relevant”:

| - Years of education they had | - Their confidence |
| --- | --- |
| - Whether they have a degree related to that specific topic | - The party they vote for |
| - Whether they have personal experience regarding that topic | - Their height |
| - Their level of intelligence in general | - Their gender |
| - Where they work | - Their age |
| - Financial incentives they might have | - Their race |
| - The recognition they received from their peers (e.g., awards, prizes) | - Their looks |
| - Their reputation among the general public | - Their sexual orientation |
| - Their moral character | - Their wealth |

### Follow-up questions about the topics:

“Please indicate how much you agree or disagree with the following statements about [topic].”

Participants rated the following items on a 5-point Agree-disagree scale:

- I have strong opinions about [topic].
- My feelings about [topic] are a reflection of my core moral beliefs and convictions.
- The topic of [topic] is politicized.

### Wordings for the topics

| **Relevance questions:** | **Follow-up questions:** |
| --- | --- |
| a new skin product | skin products |
| whether eating a specific kind of food is good or bad for you | nutrition |
| whether to invest in a stock fund | stock investments |
| abortion | abortion |
| a new policy designed to address homelessness | homelessness |
| whether a police department's response to an incident in the news was excessive | excessive use of force by  police |

## Study 2

### Expert biographies

Biographies followed the same structure:

“[Gender] has been researching [topic] for almost [Years of experience] years. [Recognition from peers] [Anecdotal experience] [Recognition from the public] [He/she] received his/her degree in [Relevance of degree] from [Prestige of institution].”

For each biography, each cue was randomly assigned to contain the values from one of the two levels. For Gender, Years of Experience, and Prestige of Institution, randomization was set up to prevent participants from seeing the same value in multiple biographies (Qualtrics file can be shared upon request).

| Cue | Level | |
| --- | --- | --- |
| **Gender** | Male:  Daniel Blake  Alex Morgan  Jordan Taylor | Female:  Maya Ellis  Nina Avery  Sophia Ellis |
| **Years of experience** | High:  13, 14, 15 | Low:  3, 4, 5 |
| **Relevance of degree** for: | High: | Low: |
| Skincare | Medicine (specializing in Dermatology) | Law |
| Nutrition | Nutrition and Dietetics | Business |
| Stock investments | Finance | Classics |
| Abortion | Medicine (specializing in OB/GYN) | Mechanical engineering |
| Police brutality | Criminology | English Literature |
| Homelessness | Social Policy | Chemistry |
| **Prestige of institution** | High:  Harvard University  Yale University  Brown University  Cornell University  Columbia University  University of Pennsylvania | Low:  Mayville State University  University of Maine at Fort Kent  University of South Alabama  University of Nevada, Reno  Del Mar College  Touro University |
| **Recognition from peers** | Present:  [He/She] has published many highly-cited articles on [topic] in peer-reviewed journals and received multiple awards from the scientific community. | Absent:  (blank) |
| **Recognition from the public** | Present:  [He/she] also has tens of thousands of followers on social media and has appeared multiple times on TV to discuss the topic. | Absent:  (blank) |
| **Anecdotal experience** for: | Present: | Absent |
| Skincare | In addition to [his/her] research, [he/she] was able to get [his/her] own chronic acne under control. | (blank) |
| Nutrition | In addition to [his/her] research, [he/she] lost 120 lbs and managed [his/her] own diabetes by changing [his/her] diet. |  |
| Stock investments | In addition to [his/her] research, [he/she] became a self-made multimillionaire before age 30. |  |
| Abortion | In addition to [his/her] research, [he/she] volunteered at a local organization that facilitates support groups for women who had abortions. |  |
| Police brutality | In addition to [his/her] research, [he/she] volunteered at the Public Safety Foundation of America. |  |
| Homelessness | In addition to [his/her] research, [he/she] volunteered at the homeless shelters in [his/her] community. |  |

Example biography:

Topic: Skincare

Gender: Female

Years of experience: Low

Recognition from peers: Present

Anecdotal experience: Present

Recognition from the public: Absent

Relevance of degree: Low

Prestige of institution: High

“Maya Ellis has been researching skincare for almost 4 years . She has published many well-cited articles on skincare in peer-reviewed journals and received multiple awards from the scientific community. In addition to her research, she was able to get her own chronic acne under control. She received her degree in Law from University of Pennsylvania.”

### Trust questions

“Based on the short biography that you just read, please indicate how much you agree or disagree with the following statements.”

Participants rated the following items on a 7-point Agree-disagree scale:

- This person is an expert on [topic].
- This person knows what they are talking about when it comes to [topic].
- I would trust this person’s opinion on [topic].
- This person is a credible source of information on [topic].

### Impressions of specific attributes

“Please answer the following questions about your impression of [name] based on their biography.”

Participants answered these questions on a 5-point scale from “None at all” to “A great deal”. Cues corresponding to each question are presented in parentheses.

- How much time did they spend studying [topic]? (Years of experience)
- How relevant is their academic background to [topic]? (Relevance of degree)
- How prestigious is the academic institution where they studied? (Prestige of institution)
- How much recognition do they receive from experts working on [topic]? (Recognition from peers)
- How much recognition do they receive from the public? (Recognition from the public)
- How much personal experience do they have when it comes to [topic]? (Anecdotal experience)

##

## Study 3

### Screening question

“When it comes to others having the right to terminate their pregnancy, are you Pro Life or Pro Choice?” (Pro-life, Pro-choice, NA/Rather not say)

### Follow-up questions

#### Confidence

“How confident are you in your views on abortion?”

Participants responded using a 5-point scale from”Not confident at all” to “Extremely confident”.

#### Belief superiority

“In your view, how much more correct are your beliefs about abortion than other people’s beliefs about this issue?”

Participants responded using a 5-point scale with the following options:

- “No more correct than other viewpoints”
- “Slightly more correct than other viewpoints”
- “Somewhat more correct than other viewpoints”
- “Much more correct than other viewpoints”
- “Totally correct (Mine is the only correct view)”

#### Moral conviction

“Please indicate how much you agree or disagree with the sentence below:

‘My feelings about abortion are a reflection of my core moral beliefs and convictions.’”

Participants responded using a 5-point Agree-Disagree scale.

##

### Biographies

Gender: Male, Expertise: High, Views: No info

Daniel Blake

Daniel Blake (born August 4, 1986) is an American researcher, author and public speaker.^[1][2]^ Blake is best known for his book on abortion titled *Abortion: What Is At Stake*.^[3]^

Education and careerBlake received his degree in Medicine from University of Arizona in 2010.^[1]^ After graduating, he started his research and publishing career in 2010. His first book, *The Three Biggest Myths About Abortion*, came out in 2014. He followed that up with *More Myths About Abortion* in 2019. He published his best-selling book, *Abortion: What Is At Stake*, in 2023.^[3]^ In addition, he has published short articles for newspapers and science blogs.

Personal life

Blake currently lives in Phoenix, Arizona.^[1]^ He married Sophia Ellis in 2018. Their son was born in 2020.

Gender: Male, Expertise: High, Views: Pro-life

Daniel Blake

Daniel Blake (born August 4, 1986) is an American researcher, author and public speaker.^[1][2]^ Blake is best known for his book on abortion titled *Abortion: What Is At Stake*.^[3]^

Education and careerBlake received his degree in Medicine from University of Arizona in 2010.^[1]^ After graduating, he started his research and publishing career in 2010. His first book, *The Three Biggest Myths About Abortion*, came out in 2014. He followed that up with *More Myths About Abortion* in 2019. He published his best-selling book, *Abortion: What Is At Stake*, in 2023.^[3]^ In addition, he has published short articles for newspapers and science blogs.

Views

Blake has been a staunch advocate of pro-life views on the topic of abortion. In his books, he has surveyed the evidence about the development of fetuses and argued that they have a right to be born.^[3]^

Gender: Male, Expertise: High, Views: Pro-choice

Daniel Blake

Daniel Blake (born August 4, 1986) is an American researcher, author and public speaker.^[1][2]^ Blake is best known for his book on abortion titled *Abortion: What Is At Stake*.^[3]^

Education and careerBlake received his degree in Medicine from University of Arizona in 2010.^[1]^ After graduating, he started his research and publishing career in 2010. His first book, *The Three Biggest Myths About Abortion*, came out in 2014. He followed that up with *More Myths About Abortion* in 2019. He published his best-selling book, *Abortion: What Is At Stake*, in 2023.^[3]^ In addition, he has published short articles for newspapers and science blogs.

Views

Blake has been a staunch advocate of pro-choice views on the topic of abortion. In his books, he has surveyed the evidence about the potential complications during pregnancy and argued that access to abortion is an essential part of women’s healthcare.^[3]^

Gender: Male, Expertise: Low, Views: No-info

Daniel Blake

Daniel Blake (born August 4, 1986) is an American researcher, author and public speaker.^[1][2]^ Blake is best known for his book on abortion titled *Abortion: What Is At Stake*.^[3]^

Education and careerBlake received his degree in Mechanical Engineering from University of Arizona in 2010.^[1]^ After working at Titan Consulting for 10 years, Blake started researching and publishing about abortion in 2020. He published his best-selling book, *Abortion: What Is At Stake*, in 2023.^[3]^ In addition, he has published short articles for newspapers and science blogs.

Personal life

Blake currently lives in Phoenix, Arizona.^[1]^ He married Sophia Ellis in 2018. Their son was born in 2020.

Gender: Male, Expertise: Low, Views: Pro-life

Daniel Blake

Daniel Blake (born August 4, 1986) is an American researcher, author and public speaker.^[1][2]^ Blake is best known for his book on abortion titled *Abortion: What Is At Stake*.^[3]^

Education and careerBlake received his degree in Mechanical Engineering from University of Arizona in 2010.^[1]^ After working at Titan Consulting for 10 years, Blake started researching and publishing about abortion in 2020. He published his best-selling book, *Abortion: What Is At Stake*, in 2023.^[3]^ In addition, he has published short articles for newspapers and science blogs.

Views

Blake has been a staunch advocate of pro-life views on the topic of abortion. In his books, he has surveyed the evidence about the development of fetuses and argued that they have a right to be born.^[3]^

Gender: Male, Expertise: Low, Views: Pro-choice

Daniel Blake

Daniel Blake (born August 4, 1986) is an American researcher, author and public speaker.^[1][2]^ Blake is best known for his book on abortion titled *Abortion: What Is At Stake*.^[3]^

Education and careerBlake received his degree in Mechanical Engineering from University of Arizona in 2010.^[1]^ After working at Titan Consulting for 10 years, Blake started researching and publishing about abortion in 2020. He published his best-selling book, *Abortion: What Is At Stake*, in 2023.^[3]^ In addition, he has published short articles for newspapers and science blogs.

Views

Blake has been a staunch advocate of pro-choice views on the topic of abortion. In his books, he has surveyed the evidence about the potential complications during pregnancy and argued that access to abortion is an essential part of women’s healthcare.^[3]^

Gender: Female, Expertise: High, Views: No info

Sophia Blake

Sophia Blake (born August 4, 1986) is an American researcher, author and public speaker.^[1][2]^ Blake is best known for her book on abortion titled *Abortion: What Is At Stake*.^[3]^

Education and careerBlake received her degree in Medicine from University of Arizona in 2010.^[1]^ After graduating, she started her research and publishing career in 2010. Her first book, *The Three Biggest Myths About Abortion*, came out in 2014. She followed that up with *More Myths About Abortion* in 2019. She published her best-selling book, *Abortion: What Is At Stake*, in 2023.^[3]^ In addition, she has published short articles for newspapers and science blogs.

Personal life

Blake currently lives in Phoenix, Arizona.^[1]^ She married Daniel Ellis in 2018. Their son was born in 2020.

Gender: Female, Expertise: High, Views: Pro-life

Sophia Blake

Sophia Blake (born August 4, 1986) is an American researcher, author and public speaker.^[1][2]^ Blake is best known for her book on abortion titled *Abortion: What Is At Stake*.^[3]^

Education and careerBlake received her degree in Medicine from University of Arizona in 2010.^[1]^ After graduating, she started her research and publishing career in 2010. Her first book, *The Three Biggest Myths About Abortion*, came out in 2014. She followed that up with *More Myths About Abortion* in 2019. She published her best-selling book, *Abortion: What Is At Stake*, in 2023.^[3]^ In addition, she has published short articles for newspapers and science blogs.

Views

Blake has been a staunch advocate of pro-life views on the topic of abortion. In her books, she has surveyed the evidence about the development of fetuses and argued that they have a right to be born.^[3]^

Gender: Female, Expertise: High, Views: Pro-choice

Sophia Blake

Sophia Blake (born August 4, 1986) is an American researcher, author and public speaker.^[1][2]^ Blake is best known for her book on abortion titled *Abortion: What Is At Stake*.^[3]^

Education and careerBlake received her degree in Medicine from University of Arizona in 2010.^[1]^ After graduating, she started her research and publishing career in 2010. Her first book, *The Three Biggest Myths About Abortion*, came out in 2014. She followed that up with *More Myths About Abortion* in 2019. She published her best-selling book, *Abortion: What Is At Stake*, in 2023.^[3]^ In addition, she has published short articles for newspapers and science blogs.

Views

Blake has been a staunch advocate of pro-choice views on the topic of abortion. In her books, she has surveyed the evidence about the potential complications during pregnancy and argued that access to abortion is an essential part of women’s healthcare.^[3]^

Gender: Female, Expertise: Low, Views: No info

Sophia Blake

Sophia Blake (born August 4, 1986) is an American researcher, author and public speaker.^[1][2]^ Blake is best known for her book on abortion titled *Abortion: What Is At Stake*.^[3]^

Education and careerBlake received her degree in Mechanical Engineering from University of Arizona in 2010.^[1]^ After working at Titan Consulting for 10 years, Blake started researching and publishing about abortion in 2020. She published her best-selling book, *Abortion: What Is At Stake*, in 2023.^[3]^ In addition, she has published short articles for newspapers and science blogs.

Personal life

Blake currently lives in Phoenix, Arizona.^[1]^ She married Daniel Ellis in 2018. Their son was born in 2020.

Gender: Female, Expertise: Low, Views: Pro-life

Sophia Blake

Sophia Blake (born August 4, 1986) is an American researcher, author and public speaker.^[1][2]^ Blake is best known for her book on abortion titled *Abortion: What Is At Stake*.^[3]^

Education and careerBlake received her degree in Mechanical Engineering from University of Arizona in 2010.^[1]^ After working at Titan Consulting for 10 years, Blake started researching and publishing about abortion in 2020. She published her best-selling book, *Abortion: What Is At Stake*, in 2023.^[3]^ In addition, she has published short articles for newspapers and science blogs.

Views

Blake has been a staunch advocate of pro-life views on the topic of abortion. In her books, she has surveyed the evidence about the development of fetuses and argued that they have a right to be born.^[3]^

Gender: Female, Expertise: Low, Views: Pro-choice

Sophia Blake

Sophia Blake (born August 4, 1986) is an American researcher, author and public speaker.^[1][2]^ Blake is best known for her book on abortion titled *Abortion: What Is At Stake*.^[3]^

Education and careerBlake received her degree in Mechanical Engineering from University of Arizona in 2010.^[1]^ After working at Titan Consulting for 10 years, Blake started researching and publishing about abortion in 2020. She published her best-selling book, *Abortion: What Is At Stake*, in 2023.^[3]^ In addition, she has published short articles for newspapers and science blogs.

Views

Blake has been a staunch advocate of pro-choice views on the topic of abortion. In her books, she has surveyed the evidence about the potential complications during pregnancy and argued that access to abortion is an essential part of women’s healthcare.^[3]^

### Comprehension checks

#### Researcher’s college major

“According to [name]'s biography that you just read, what discipline did [he/she] study in college?”

Participants responded using the following options:

- Medicine
- Nutrition and Dietetics
- Mechanical Engineering
- Business

#### Researcher’s views

“Based on the biography you read, what do you think [name]’s views are when it comes to abortion?”

Participants responded using the following options:

- Blake advocates for pro-choice views
- Blake advocates for pro-life views
- Don’t know / Not sure

# Supplementary tables and figures

## Figure S1

Effect of the researcher’s gender on trust by participant’s views on abortion (Study 3)


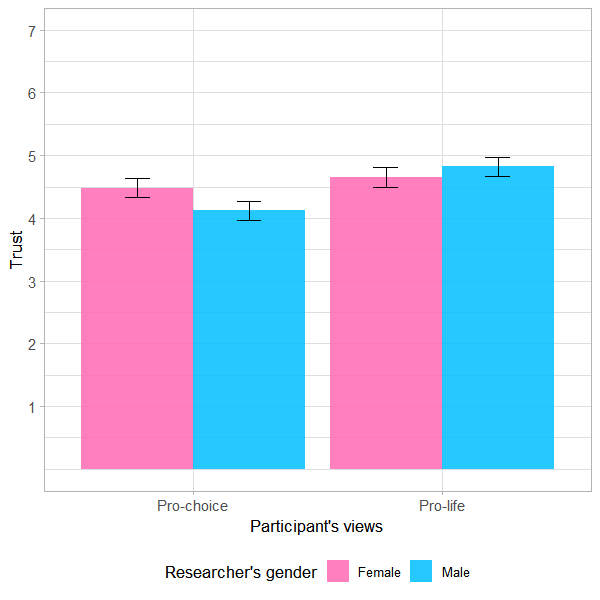


## Table S1

Impressions of experts’ attributes predicting trust (Study 2)

| Intercept | 0.001  (0.031) |
| --- | --- |
| Time spent researching | 0.291  (0.013)*** |
| Relevance of academic background | 0.214  (0.012)*** |
| Prestige of institution | 0.055  (0.012)*** |
| Recognition from peers | 0.226  (0.014)*** |
| Recognition from public | 0.078  (0.019)*** |
| Anecdotal experience | 0.226  (0.015)*** |
| *R^2^* (fixed-effects) | 0.521 |
| *R^2^* (total) | 0.659 |
| ICC | 0.3 |
| *Note.* p < 0.1, * p < 0.05, ** p < 0.01, *** p < 0.001. Estimates are standardized. | |

## Table S2

Effects of cues of expertise on impressions of experts’ attributes (Study 2)

| Cue | Time spent studying | Relevance of background | Peer recognition | Personal experience | Prestige of inst. | Public recognition |
| --- | --- | --- | --- | --- | --- | --- |
| More research experience | 1.188 (0.024)*** | 0.142 (0.023)*** | 0.235 (0.026)*** | 0.357 (0.027)*** | 0.088 (0.026)*** | 0.148 (0.026)*** |
| Relevant degree | 0.197 (0.025)*** | 1.478 (0.023)*** | 0.229 (0.028)*** | 0.28 (0.029)*** | 0.179 (0.027)*** | 0.125 (0.027)*** |
| Recognition from peers | 0.102 (0.026)*** | 0.093 (0.024)*** | 1.101 (0.028)*** | 0.132 (0.029)*** | 0.056 (0.028)* | 0.422 (0.027)*** |
| Anecdotal experience | 0.027 (0.026) | -0.005 (0.024) | 0.053 (0.028). | 0.454 (0.029)*** | 0.025 (0.028) | 0.115 (0.027)*** |
| Degree from a prestigious institution | 0.035 (0.024) | 0.05 (0.023)* | 0.109 (0.026)*** | 0.022 (0.027) | 1.23 (0.026)*** | 0.094 (0.026)*** |
| Recognition from public | 0.023 (0.026) | 0.026 (0.024) | 0.217 (0.028)*** | 0.112 (0.029)*** | 0.006 (0.028) | 1.041 (0.027)*** |
| Being female | 0.015 (0.024) | 0.03 (0.023) | 0.026 (0.026) | 0.057 (0.027)* | 0.04 (0.026) | -0.005 (0.026) |
| *Note.* p < 0.1, * p < 0.05, ** p < 0.01, *** p < 0.001. Estimates are standardized. | | | | | | |

## Table S3

Effects of the researcher’s expertise, views, and gender on trust (Study 3)

|  | *df* | *η^2^_G_* | *F* | *p* |
| --- | --- | --- | --- | --- |
| Expertise | 1 | .075 | 142.09 | < .001 |
| Researcher’s views | 2 | .195 | 213.06 | < .001 |
| Researcher’s gender | 1 | .002 | 3.45 | .063 |
| Expertise x Researcher’s views | 2 | .001 | 1.24 | .289 |
| Expertise x Researcher’s gender | 1 | .001 | 1.64 | .201 |
| Researcher’s views x Researcher’s gender | 2 | .001 | 0.96 | .383 |
| Expertise x Researcher’s views x Researcher’s gender | 2 | .001 | 0.89 | .412 |
| Residual | 1764 |  |  |  |

## Table S4

Effects of the researcher’s expertise, views, and gender on individual trust items (Study 3)

|  |  | “Is an expert” | | “Knows what they are talking about” | | “I would trust their opinion” | | “Is a credible source of information” | |
| --- | --- | --- | --- | --- | --- | --- | --- | --- | --- |
|  | *df* | *F (p)* | *η^2^_G_* | *F (p)* | *η^2^_G_* | *F (p)* | *η^2^_G_* | *F (p)* | *η^2^_G_* |
| Expertise | 1 | 156.27  (<.001) | 0.088 | 94.78  (<.001) | 0.058 | 71.77  (<.001) | 0.047 | 113.29  (<.001) | 0.067 |
| Views | 2 | 122.84  (<.001) | 0.122 | 183.36  (<.001) | 0.172 | 278.55  (<.001) | 0.24 | 163.58  (<.001) | 0.156 |
| Gender | 1 | 2.79  (0.095) | 0.002 | 3.62  (0.057) | 0.002 | 1.87  (0.171) | 0.001 | 3.29  (0.070) | 0.002 |
| Expertise x Views | 2 | 0.24  (0.788) | 0 | 1.77  (0.170) | 0.002 | 1.16  (0.312) | 0.001 | 1.70  (0.183) | 0.002 |
| Expertise x Gender | 1 | 0.58  (0.446) | 0 | 3.92  (0.048) | 0.002 | 1.34  (0.247) | 0.001 | 1.12  (0.290) | 0.001 |
| Views x Gender | 2 | 1.29  (0.275) | 0.001 | 1.11  (0.331) | 0.001 | 0.61  (0.543) | 0.001 | 1.06  (0.348) | 0.001 |
| Expertise x Views x Gender | 2 | 0.69  (0.504) | 0.001 | 1.75  (0.174) | 0.002 | 0.17  (0.842) | 0 | 1.15  (0.317) | 0.001 |
| Residuals | 1764 | |  |  |  |  |  |  |  |

## Table S5

Effects of the researcher’s expertise, views, and gender on trust, by participant’s views (Study 3)

|  | *df* | *η^2^_G_* | *F* | *p* |
| --- | --- | --- | --- | --- |
| Expertise | 1 | .077 | 145.97 | < .001 |
| Researcher’s views | 2 | .199 | 218.87 | < .001 |
| Researcher’s gender | 1 | .002 | 3.54 | .060 |
| Participant’s views | 1 | .020 | 36.43 | < .001 |
| Expertise x Researcher’s views | 2 | .002 | 1.60 | .203 |
| Expertise x Researcher’s gender | 1 | .001 | 1.62 | .203 |
| Researcher’s views x Researcher’s gender | 2 | .001 | 0.80 | .449 |
| Expertise x Participant’s views | 1 | .001 | 0.93 | .336 |
| Researcher’s views x Participant’s views | 2 | .002 | 1.72 | .179 |
| Researcher’s gender x Participant’s views | 1 | .007 | 12.23 | < .001 |
| Expertise x Researcher’s views x Researcher’s gender | 2 | .001 | 0.83 | .435 |
| Residual | 1759 |  |  |  |

## Table S6

Individual differences moderating the effect of the researcher’s views on trust (Study 3)

|  | |  | | Predictor | |  | |
| --- | --- | --- | --- | --- | --- | --- | --- |
|  | | Confidence | | Moral conviction | | Superiority | |
|  | *df* | *F* | *p* | *F* | *p* | *F* | *p* |
| Expertise | 1 | 132.33 | < .001 | 117.01 | < .001 | 131.50 | < .001 |
| Views | 2 | 230.26 | < .001 | 218.63 | < .001 | 225.35 | < .001 |
| Predictor | 1 | 36.50 | < .001 | 3.07 | 0.080 | 6.53 | 0.021 |
| Expertise x Views | 2 | 1.11 | 0.574 | 1.86 | 0.468 | 1.25 | 0.574 |
| Expertise x Predictor | 1 | 0.00 | 0.990 | 1.33 | 0.499 | 4.21 | 0.121 |
| Views x Predictor | 2 | 51.00 | < .001 | 28.18 | < .001 | 47.74 | < .001 |
| Expertise x Views x Predictor | 2 | 0.07 | 1.000 | 2.37 | 0.281 | 0.28 | 1.000 |
| Residuals | 1764 |  |  |  |  |  |  |

*Note.* P-values were adjusted using the Holm correction.

## Table S7

Effects of the researcher’s expertise, views, and gender on impressions of the researcher’s attributes (Study 3)

|  |  | Years of experience | | Relevance | | Prestige | | Peer recognition | | Public recognition | | Personal experience | |
| --- | --- | --- | --- | --- | --- | --- | --- | --- | --- | --- | --- | --- | --- |
|  | *df* | *F (p)* | *η^2^_G_* | *F (p)* | *η^2^_G_* | *F (p)* | *η^2^_G_* | *F (p)* | *η^2^_G_* | *F (p)* | *η^2^_G_* | *F (p)* | *η^2^_G_* |
| Expertise | 1 | 249.87  (<.001) | 0.129 | 690.79  (<.001) | 0.286 | 27.04  (<.001) | 0.016 | 73.04  (<.001) | 0.042 | 27.77  (<.001) | 0.017 | 71.73  (<.001) | 0.042 |
| Views | 2 | 45.44  (<.001) | 0.049 | 28.33  (<.001) | 0.031 | 20.15  (<.001) | 0.022 | 24.64  (<.001) | 0.027 | 13.60  (<.001) | 0.015 | 31.13  (<.001) | 0.034 |
| Gender | 1 | 1.29  (0.256) | 0.001 | 0.13  (0.716) | 0 | 0.35  (0.553) | 0 | 0.05  (0.823) | 0 | 0.69  (0.406) | 0 | 4.73  (0.030) | 0.003 |
| Expertise x Views | 2 | 2.37  (0.094) | 0.003 | 5.88  (0.003) | 0.007 | 0.76  (0.468) | 0.001 | 0.24  (0.790) | 0 | 0.24  (0.787) | 0 | 1.39  (0.250) | 0.002 |
| Expertise x Gender | 1 | 0.95  (0.330) | 0.001 | 0.22  (0.637) | 0 | 0.00  (0.959) | 0 | 0.00  (0.962) | 0 | 0.00  (0.946) | 0 | 0.16  (0.691) | 0 |
| Views x Gender | 2 | 0.34  (0.709) | 0 | 0.00  (0.998) | 0 | 0.86  (0.422) | 0.001 | 0.28  (0.759) | 0 | 0.06  (0.943) | 0 | 0.05  (0.947) | 0 |
| Expertise x Views x Gender | 2 | 0.04  (0.961) | 0 | 0.12  (0.886) | 0 | 0.48  (0.621) | 0.001 | 1.40  (0.246) | 0.002 | 0.21  (0.810) | 0 | 0.85  (0.428) | 0.001 |
| Residuals | 1764  (1763 for Years of experience) | |  |  |  |  |  |  |  |  |  |  |  |

## Table S8

Individual differences and the researcher’s expertise predicting projection of one’s own views on the researcher (Study 3)

|  | Predictor | | |
| --- | --- | --- | --- |
|  | Confidence | Moral conviction | Belief superiority |
| Intercept | -0.737***  (0.124) | -0.743***  (0.124) | -0.748***  (0.125) |
| Expertise (High) | 0.053  (0.178) | 0.065  (0.178) | 0.069  (0.179) |
| Predictor | -0.046  (0.125) | 0.107  (0.133) | 0.175  (0.125) |
| Expertise x Predictor | -0.066  (0.176) | -0.147  (0.180) | 0.021  (0.182) |
| N | 573 | 573 | 573 |
| *R^2^* | 0.00 | 0.00 | 0.01 |
| *Note.* Analyses were performed on participants who did not receive information about the researcher’s views (i.e., those assigned to the No-information condition). P-values were adjusted using the Holm correction. *p* < 0.1, * *p* < 0.05, ** *p* < 0.01, *** *p* < 0.001. | | | |

## Table S9

Multinomial regression on participants’ guesses about the researcher’s views (Study 3)

|  | log(OR) | 95% CI | *p* |
| --- | --- | --- | --- |
| Pro-life (vs. “Don’t know”) | | | |
| Expertise (High) | -0.02 | [-0.57, 0.54] | >0.9 |
| Gender (Male) | 0.44 | [-0.07, 0.94] | 0.089 |
| Expertise x Gender | 0.02 | [-0.74, 0.78] | >0.9 |
| Pro-choice (vs “Don’t know) | | | |
| Expertise (High) | 0.46 | [-0.12, 1.0] | 0.12 |
| Gender (Male) | -0.47 | [-1.1, 0.19] | 0.2 |
| Expertise x Gender | 0.43 | [-0.47, 1.3] | 0.3 |
| *Note.* Analysis was performed on participants who did not receive information about the researcher’s views (i.e., those assigned to the No-information condition). | | | |
